# The Intersection of Dementia-Friendly Initiatives and Age-Friendly Environments: The Integration Models

**DOI:** 10.1093/geroni/igab046.1840

**Published:** 2021-12-17

**Authors:** Fei Sun, Ha Neul Kim, Lucas Prieto, Stéfanie Fréel, katrin Seeher, Huali Wang

**Affiliations:** 1 Michigan State University, East Lansing, Michigan, United States; 2 World Health Organization, Geneva, Geneve, Switzerland; 3 Peking University Institute of Mental Health (Sixth Hospital), Beijing, Beijing, China (People's Republic)

## Abstract

While age friendly city (AFC) initiatives aim to build supportive physical and social environments for older adults, dementia-friendly initiatives (DFI) see the critical need of persons living with dementia (PWD) to be included in society. Given the close relationship between advanced age and dementia risk, communities facing challenges of aging and dementia will benefit from the integration of DFI and AFC. This study aims to summarize the differences between AFC and DFI practice and to identify integrative models for DFI and AFC based upon cases in the U.S.A and China. Qualitative interviews with 11 stakeholders from Massachusetts and Michigan of the U.S.A. and Beijing and Shanghai in mainland China were recorded via Zoom and transcribed for analyses in order to identify different integration models. A summary of differences and commonalities between AFC and DFI core values, key players, major activities, and outcomes is reported. Four practice models of AFC and DFI based upon case analyses were described as sequential integration, concurrent integration, sequential separation, and concurrent separation. Massachusetts’ model is unique in the support from the state government to integrate both from the beginning, and Michigan witnessed separate efforts between grassroots-based agencies and the state government. Shanghai model represents a sequential integration that includes DFI in local government’s long-term aging policy plan, while AFC and DFI in Beijing have a loose connection despite progress made for each initiative. Communities need to develop a practice model considering its local community needs, policy support, and sustainable resources available.

